# Repurposing thioridazine for inducing immunogenic cell death in colorectal cancer via eIF2α/ATF4/CHOP and secretory autophagy pathways

**DOI:** 10.1186/s12964-023-01190-5

**Published:** 2023-07-24

**Authors:** Thu-Ha Tran, Ming Kao, Hsiao-Sheng Liu, Yi-Ren Hong, Yeu Su, Chi-Ying F. Huang

**Affiliations:** 1 Taiwan International Graduate Program in Molecular Medicine, National Yang Ming Chiao Tung University and Academia Sinica, Taipei, 112 Taiwan; 2 Institute of Biopharmaceutical Sciences, National Yang Ming Chiao Tung University, Taipei, 112 Taiwan; 3 Department of Microbiology and Immunology, College of Medicine, National Cheng Kung University, Tainan, 701 Taiwan; 4 Center for Cancer Research, College of Medicine, Kaohsiung Medical University, Kaohsiung, 807 Taiwan; 5 M. Sc. Program in Tropical Medicine, College of Medicine, Kaohsiung Medical University, Kaohsiung, 807 Taiwan; 6 Graduate Institutes of Medicine, College of Medicine, Kaohsiung Medical University, Kaohsiung, 807 Taiwan; 7 Department of Biological Sciences, National Sun Yat-Sen University, Kaohsiung, 804 Taiwan; 8 Department of Medical Research, Kaohsiung Medical University Hospital, Kaohsiung, 807 Taiwan; 9 Neuroscience Research Center, Kaohsiung Medical University, Kaohsiung, 807 Taiwan; 10 Department of Biochemistry, College of Medicine, Kaohsiung Medical University, Kaohsiung, 807 Taiwan; 11 Institute of Clinical Medicine, College of Medicine, National Yang Ming Chiao Tung University, Taipei, 112 Taiwan; 12 Department of Biotechnology and Laboratory Science in Medicine, National Yang Ming Chiao Tung University, Taipei, 112 Taiwan

**Keywords:** Colorectal cancer, Thioridazine, Immunogenic cell death, Endoplasmic reticulum stress, Secretory autophagy

## Abstract

**Background:**

Colorectal cancer (CRC) is a highly prevalent cancer type with limited targeted therapies available and 5-year survival rate, particularly for late-stage patients. There have been numerous attempts to repurpose drugs to tackle this problem. It has been reported that autophagy inducers could augment the effect of certain chemotherapeutic agents by enhancing immunogenic cell death (ICD).

**Methods:**

In this study, we employed bioinformatics tools to identify thioridazine (THD), an antipsychotic drug, and found that it could induce autophagy and ICD in CRC. Then in vitro and in vivo experiments were performed to further elucidate the molecular mechanism of THD in CRC.

**Results:**

THD was found to induce endoplasmic reticulum (ER) stress in CRC cells by activating the eIF2α/ATF4/CHOP axis and facilitating the accumulation of secretory autophagosomes, leading to ICD. In addition, THD showed a remarkable ICD-activating effect when combined with oxaliplatin (OXA) to prevent tumor progression in the mouse model.

**Conclusions:**

Together, our findings suggest that the repurposed function of THD in inhibiting CRC involves the upregulation of autophagosomes and ER stress signals, promoting the release of ICD markers, and providing a potential candidate to enhance the clinical outcome for CRC treatment.

Video Abstract

**Supplementary Information:**

The online version contains supplementary material available at 10.1186/s12964-023-01190-5.

## Introduction

Colorectal cancer (CRC) is the leading cancer worldwide in terms of incidence and mortality, with approximately 147 000 occurrences and 53 200 deaths a year, in the US alone in 2020 [1]. Although the prognosis of metastatic CRC (mCRC) has improved recently, only limited effective targeted therapy drugs are available. Moreover, the results of most clinical trials for immune checkpoint inhibitors (ICIs) were discouraging. The treatment for CRC is especially challenging owing to the highly frequent genomic alterations during disease progression. In this regard, *Adenomatous Polyposis Coli (APC)* mutation has been identified to occur initially, accounting for approximately 80% of CRC, which converts the normal epithelium into a hyperplastic one. This is followed by the sequential mutations of *KRAS*, *SMAD4*, *TP53,* and other unknown exacerbating invasion and metastasis [2]. Therefore, finding an effective remedy targeting crucial genetic aberrations is necessary to improve drug response and inhibit tumor progression for CRC.

The emergence of abnormal cells activates the immune system’s surveillance which can then recognize and eliminate them. However, malignant cells could escape immune surveillance by down-regulating tumor antigens, up-regulating pro-survival genes, or developing an immunosuppressive tumor microenvironment [3]. Therefore, immunotherapy was developed to target the immune checkpoint. For example, the programmed death receptor 1 (PD-1) inhibitor can regulate T cell-mediated immunity and improve the immune response against cancer [4]. However, ICIs show resistance in a plenty of solid tumor patients, and treatment efficacy in CRC patients is reduced [5, 6]. In addition, CRC cells are categorized into microsatellite stability (MSS) or microsatellite instability (MSI) depending on the deficiency of mismatch-repair genes. Although the objective response rate of immunotherapy in the MSI patient group is encouraging at 40%, there was 0% response in MSS patients [7]. Another potential strategy for boosting the immune response to eliminate cancer cells is inducing immunogenic cell death (ICD). The deaths of cancer cells in such a scenario lead to the release of pro-inflammatory cytokines, activating cytotoxic T lymphocytes which can kill cancer cells and elicit an antitumor immune response. In brief, inducing ICD in cancer cells is similar to a type of “cancer vaccine,” a promising cancer treatment strategy [8].

ICD is a cell death mechanism that can activate the adaptive immune response in hosts with a competent immune system. Induction of cellular stress leads to the release of damage-associated molecular patterns (DAMPs), a hallmark of ICD, to recruit antigen-presenting cells including macrophages, dendritic cells (DC), and neutrophils. Upon receiving the signal, mature antigen-presenting cells move to lymph nodes to activate T cells and drive the cytotoxic immune response [9]. The first DAMPs to occur in ICD are calreticulin (CRT) [10]. CRT translocating to the plasma membrane marks the beginning of phagocytosis of DC and the presentation of tumor antigen. Subsequently, extracellular adenosine triphosphate (ATP) is released, promoting the maturation of DC and macrophage, as well as facilitating cytokine synthesis. Next, high mobility group box 1 (HMGB1) is secreted in the mid-late-stage of apoptosis and functions as the activator of toll-like receptor 4 (TLR4), presenting MHC class I peptides to CD8^+^ T cells, following which Annexin A1 (ANXA1) binds to formyl peptide receptor 1 (FPR1) on DC, leading to the interaction of dying cells and antigen-presenting cells [10]. ICD can be induced by several types of stresses, including pathogens infection, immunogenic chemotherapy, radiation therapy, hypericin-based photodynamic therapy, high hydrostatic pressure, and necroptosis leading to autophagy, inflammation, TLR signal, HMGB1 release, or unfolded protein response. Many drugs have been proven as ICD inducers, for example, anthracycline (doxorubicin and mitoxantrone), cardiac glycosides (digoxin), oxaliplatin (OXA), bleomycin and cyclophosphamide [11].

Several autophagy inducers have been documented to induce ICD such as rapamycin, in renal cancer carcinoma and malignant glioma cells [12, 13], and thiostrepton (TST) in various solid tumor cell lines [14]. Autophagy regulates ATP and HMGB1 release, including the recruitment of antigen-presenting cells to induce ICD [15]. Besides, autophagy could enhance the immune response by preventing the death of CD4^+^ T cells [16]. However, autophagy inducers often work together with an ICD-inducing drug to obtain a desirable outcome since distinct drugs might function through different pathways to activate the release of DAMPs, induce ICD, and generate an antitumor effect. Even though the cytotoxic intensity is dependent on the immune response, combined treatment does have the advantage to induce true ICD [8].

To prevent the unexpected adverse effects from novel compounds, in this study we selected FDA-approved drugs with more comprehensive safety and side effects for repurposing toward autophagy induction. Through bioinformatics analysis, thioridazine (THD), an FDA-approved antipsychotic drug, is predicted to have the potential for ICD induction since it shares common features with drugs that are known to induce autophagy and ICD. In addition, THD has been reported to repress the Wnt/β-catenin signaling pathway in glioblastoma [17, 18], a major perturbed pathway in CRC, and elicit a cytotoxic effect in lung cancer stem cells [19] and colorectal stem cells [20].

In this study, we provide evidence that THD would be beneficial for the treatment of CRC, by elucidating the effect of THD on inducing ER stress and autophagy in CRC cells, suggesting a subsequent induction of ICD when combined with chemotherapy. We also examined the effect of THD on apoptosis induction in CRC cells to provide an extensive understanding of the anticancer mechanisms of THD in CRC.

## Materials & methods

### Cell culture and chemicals

This study has employed CRC cell lines including HT29, LoVo, HCT116, and RKO kindly gifted from Dr. Michael Hsiao’s laboratory in the Genomics Research Center of Academia Sinica, Taiwan. Mouse CRC cell line CT26 was purchased from Bioresource Collection and Research Center (#60,443). SW480 ATG5^+/+^ and SW480 ATG5^−/−^ cells were kindly gifted from Dr. Hsiao-Sheng Liu in National Cheng Kung University. HT29 and HCT116 cells were cultured in McCoy's 5a medium, while RKO, LoVo, CT26, and SW480 cells were cultured in EMEM, F12K, and RPMI 1640, and DMEM media, respectively. All the cells were cultured in media supplemented with 10% fetal bovine serum (FBS) and 1% penicillin–streptomycin in an incubator containing 5% CO_2_ at 37 °C.

For the cell culture experiment, 20 mM of THD, 20 mM of TST, and 10 mM of 5-fluorouracil were prepared in dimethyl sulfoxide (DMSO) solvent. The solution of 5 mM oxaliplatin was prepared in a water solvent.

### Bioinformatics analysis

The microarray data of autophagy inducers including TST, sirolimus, torin-1, and simvastatin in CRC cell HT29 were queried in the CLUE database (http://clue.io). Top similar compounds with a score higher than 85 were selected to find the common list that is potential for inducing autophagy and ICD in CRC cells.

### Sulforhodamine B (SRB) assay

CRC cancer cells were seeded at a density of 4 000 cells per well in 96-well plates, drugs were treated 16 h after seeding. After the drug treatment for 24, 48, and 72 h, cells were fixed at 4 °C for 1 h with 10% trichloroacetic acid, then washed and followed by SRB staining for 1 h. Then the wells were washed with 1% acetic acid before the solubilization of SRB dye by 10 mM Tris-base. The absorbance was detected with a microplate reader at 510 nm wavelength.

### Clonogenic assay

Cells were seeded in 6-well plates with a density of 400 cells per well for 10 to 14 days. Drugs were added 24 h afterward. The culture medium containing tested drugs was changed every three days. For the sequential combination treatment, 1 μM of THD was treated for 3 days before completely washed out, followed by 5-FU or OXA treatment for 7 days with a concentration of 0.5 μM. After the treatment, PBS was used to wash the cells, followed by 0.5% crystal violet staining for 1 h. After the crystal violet removal and water rinsing, the colonies were counted via ImageJ software.

### Western blot

Cells were seeded at the density of 6–8 × 10^5^ cells in 6 cm dishes, followed by drug treatment according to the experimental design. To obtain total cell lysate, RIPA lysis buffer was used, supplemented with protease inhibitor tablets (Roche Diagnostic AB, Stockholm, Sweden). Lysates were resolved by SDS-PAGE and electro-transferred onto the PVDF membrane (Millipore). Proteins on the membranes were then incubated overnight with primary antibodies, then the horseradish peroxidase (HRP)-conjugation secondary antibodies were incubated for 1 h. The protein expression was detected with the chemiluminescence (ECL™) method, images were taken by a Luminescence Imaging System (LAS-4 000™, Fuji Photo Film CO., Ltd).

### Flow cytometry for calreticulin detection

Cells were seeded in a 6-well plate for 24 h before drug treatment. After a 24-h drug treatment, 1X TrypLE TM express enzyme (Gibco®) was added to collect the cells. Then cells were incubated with anti-calreticulin antibody (ab196159, Abcam) diluted at the ratio 1:200 in 2% FBS RPMI medium for 1 h at 4 °C. After being washed with PBS two times, cells were resuspended in 0.3 ml PBS before the detection via flow cytometry (FACS Calibur TM, BD). Calreticulin exposure level was analyzed via FlowJo software.

### Extracellular adenosine triphosphate (ATP) detection

Cells were seeded in a 96-well plate at the density of 10 000 cells per well. Drugs and reagents provided by the kit were added 24 h after cell seeding. Extracellular ATP levels were detected by the ENLITEN® ATP detection kit, following the manufacturer’s protocol. The luminescence signal was measured by an Infinite 200 pro plate reader (TECAN).

### Extracellular HMGB1 analysis

Supernatants of the samples were collected to determine the level of extracellular HMGB1 using an HMGB1 ELISA kit (#6010, Chrondrex), following the manufacturer’s protocol. The absorbance was measured by Infinite 200 Pro plate reader (TECAN) at 450 nm.

### Animal study

Male Balb/c mice, aged 3 weeks, were received from National Laboratory Animal Center (Taipei, Taiwan). A week after obtaining the mice, 12 mice were separated randomly into four groups and subcutaneously injected with drug-treated CT26 cells. For tumor vaccination assay, CT26 cells were treated with OXA, OXA + THD, or OXA + TST for 24 h, following which 1.5 × 10^6^ cells were resuspended in a solution of 50% Matrigel (BD Biosciences, Franklin Lakes, NJ). Phosphate-buffered saline was used in the control group. After 7 days, 1 × 10^6^ naïve CT26 cells in Matrigel were injected into the left side as a challenge. The body weight and tumor size were monitored every 3 days. Tumor size was measured with an electronic caliper to calculate tumor volume by the formula: volume = 0.5 × length × width^2^.

### Lentivirus knockdown of ATG7

The shRNA to knockdown *ATG7* was purchased from RNAi Core, Academia Sinica, Taiwan (TRCN0000375444). To construct and amplify the lentivirus, serum-free DMEM medium containing PolyJetTM (SignaGen Laboratories, SL100688) was mixed with a solution of *ATG7* shRNA-carrying plasmid, pCL packaging plasmid, and PMDG envelope plasmid to form PolyJet/DNA complex at room temperature. The mixture was then added to HEK293T cells seeded in a 6 cm dish at 37℃ for 16 h. The serum-free DMEM was then replaced with DMEM medium supplemented with 1% BSA. After that, the viruses-containing medium was collected 24 and 48 h post medium change and stored at -80℃. To transfect the lentivirus, CT26 cells were seeded at the density of 8 × 10^5^ cells in a 6-well plate. Then virus-containing medium was added and incubated for 24 h before the medium was replaced with a medium containing 10 mg/ml puromycin to select the successfully transfected cells.

### Extracellular vesicles measurement

HT29 cells were seeded at the density of 2 × 10^6^ cells in 10 cm dishes, followed by THD treatment for 24 h. The concentration of extracellular vesicles was measured using the NanoSight NS300 instrument (Malvern Instruments Ltd, Malvern, UK) equipped with a 488 nm laser and CCD camera (sCMOS). Data were analyzed using the Nanoparticle Tracking Analysis (NTA) software (version 3.1 builds 3.1.46). The syringe pump speed was set to 30. Readings were taken in a single capture during the 60 s at 25 frames per second (fps), with the camera level set to 15 and manual monitoring of temperature.

### Caspase activity assay

The enzymatic activities of caspases 3, 8, and 9 were quantified with the kit of caspase 3, 8, and 9 fluorometric assays (BioVision Inc., Milpitas, CA, USA), respectively. The cells were seeded in 6 cm dishes overnight, followed by drug treatment. Cells were then harvested and lysed to measure protein concentration. Each sample contains 100 µg total protein, and the volume was adjusted with cell lysis buffer to make 50 µL. Then 50 µL Reaction Buffer containing 10 mM DTT was added and transferred to a 96-well black plate. After that, 5 µL of the 1 mM DEVD-, IETD-, or LEHD-AFC substrates for caspase 3, 8, and 9 activities, respectively was added and incubated for 1 h at 37 °C. Finally, the fluorescence signals were measured by a microplate reader (Infinite 200 PRO) with an excitation/emission filter of 400/505 nm.

### Statistical analysis

All statistical analysis was performed using the student's *t*-test to calculate the statistical significance. *: *p* < 0.05; **: *p* < 0.01; ***: *p* < 0.001.

## Results

### Dopamine receptor antagonist thioridazine can induce autophagy in human CRC cells

**Fig. 1:**
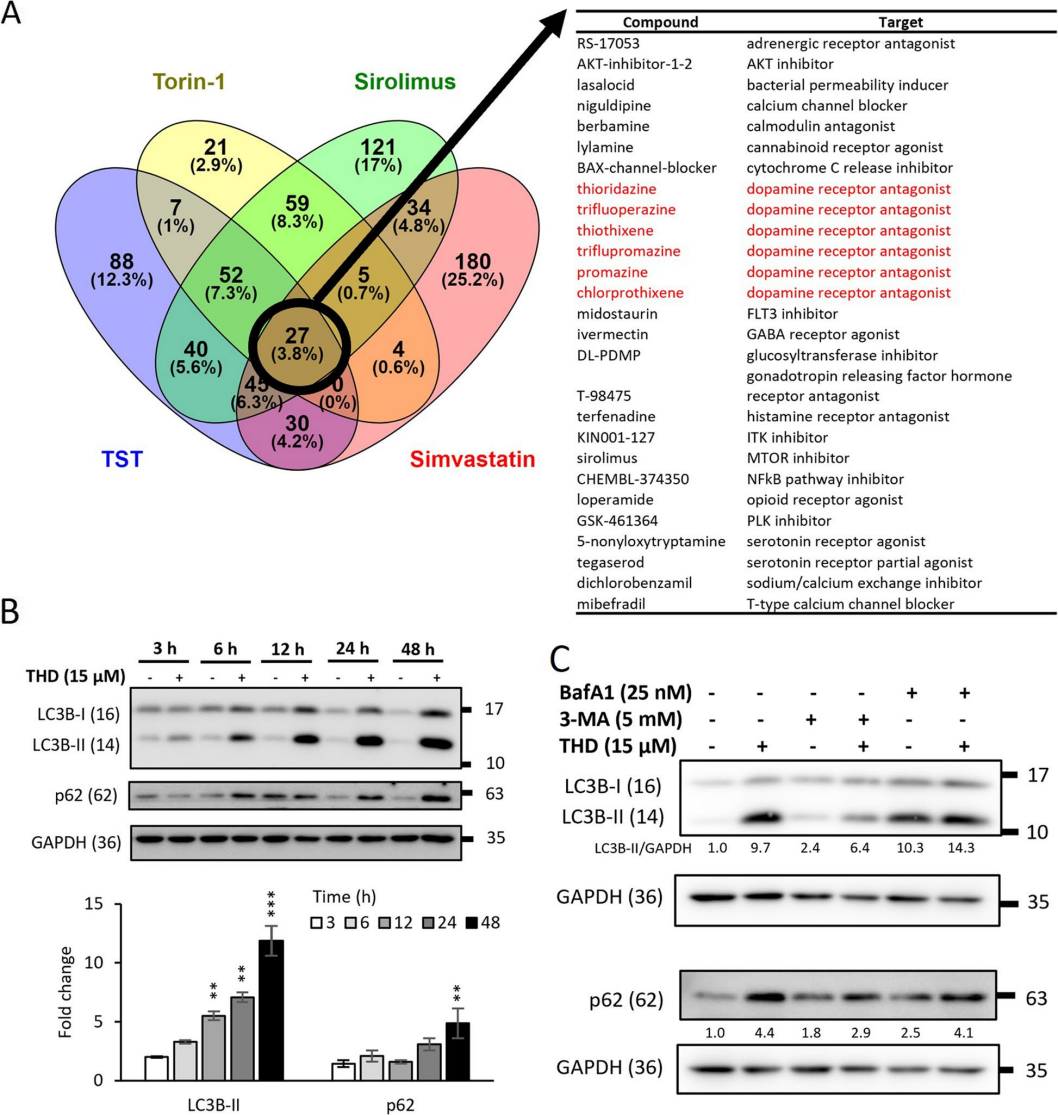
Identification and validation of thioridazine as an autophagy inducer in human CRC cells. **A** The gene signatures in HT29 human CRC cells following treatment with four autophagy inducers-TST (thiostrepton), torin-1, sirolimus (rapamycin), and simvastatin in HT29 were analyzed (left) to find 27 compounds with similar activities (a score higher than 85) (right). **B** HT29 cells were treated with 15 µM THD for varying lengths of time (3, 6, 12, 24, and 48 h) and expression of two autophagy markers, LC3B-II and p62, were analyzed by western blotting. **C** Protein levels of LC3B-II and p62 in HT29 cells following 15 µM THD treatment in the absence or presence of 3-MA (5 mM) and BafA1 (25 nM) for 6 h were analyzed by western blotting. GAPDH was used as the internal control in (**B**) and (**C**). Data are mean ± SD (*N* = 3). *: *p* < 0.05; **: *p* < 0.01; ***: *p* < 0.001

We addressed the ICD-inducing properties of autophagy inducers by searching for autophagy-inducing effect in FDA-approved drugs to repurpose them for potential ICD induction. The strategy was to first identify the shared genetic effect of autophagy inducers. Toward this end, the microarray data of four well-known autophagy inducers thiostrepton (TST), torin-1, sirolimus (rapamycin), and simvastatin were retrieved from the CLUE database. Subsequently, the lists of compounds that exhibited similar gene signatures to these compounds when treated on HT29 human CRC cells were overlapped to identify the common compounds. Among 27 compounds identified, 6 were dopamine receptor antagonists, suggesting their potential for inducing autophagy-ICD in CRC cells (Fig. 1A). A comparison of the in vitro cytotoxicity of several dopamine receptor antagonists in various human CRC cells revealed thioridazine (THD) to be the most potent effector of cytotoxicity on CRC cells (Supplementary Figure S1). Therefore, we selected THD for further investigation. The autophagy-inducing effect of THD is demonstrated in HT29 and other human CRC cells through a time-dependent increase of autophagy markers LC3B-II and p62 following drug treatment (Fig. 1B & Supplementary Figure S2A). In a subsequent experiment, the autophagy-inducing potential of THD was examined in the presence of either 3-methoxyamphetamine (3-MA), an inhibitor for the early-stage autophagy through the suppression of autophagosome formation, or bafilomycin A1 (BafA1), the late-stage autophagy inhibitor that blocks the binding between lysosome and autophagosome. We found that while the levels of both markers induced by THD in these cells were markedly reduced by 3-MA co-treatment, no significant change in either marker was detected when cells were co-treated with BafA1 (Fig. 1C & Supplementary Figure S2B). These results suggested that THD could potentially hinder the degradation of autophagosome during late-stage autophagy, similar to the effect of BafA1, besides inducing autophagosome formation, leading to an accumulation of autophagosome inside cells, consistent with the constant increase of LC3B-II and p62 from 3 to 48 h of THD treatment as shown in Fig. 1B.

### Thioridazine induces ER stress and ICD markers in human CRC cells

**Fig. 2:**
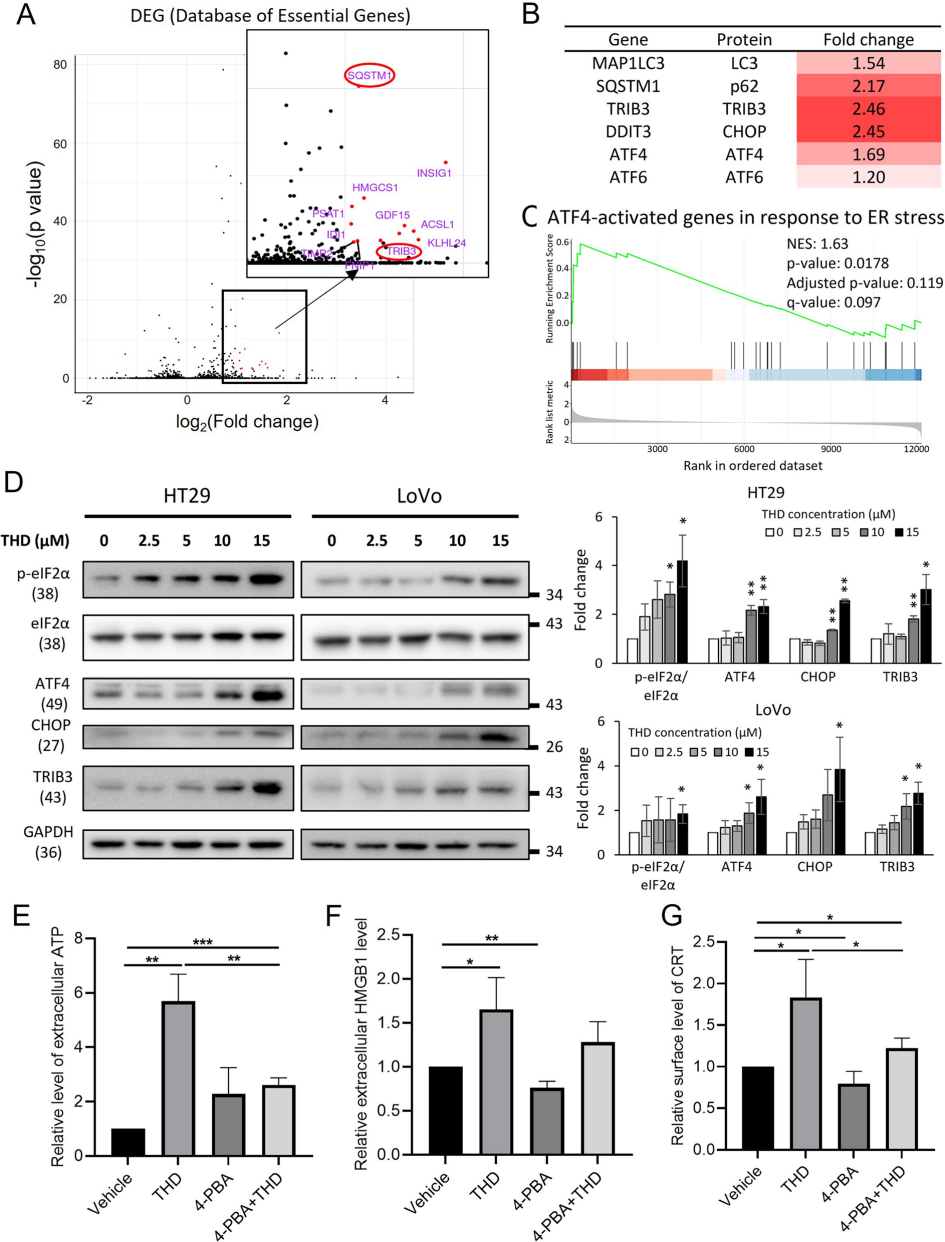
Thioridazine induces ER stress and ICD markers in CRC cell lines. Next-generation sequencing (NGS) of total RNA following THD treatment for 6 h was performed in HT29 cells. **A** Number of upregulated genes. **B** Autophagy and ER stress markers. **C** GSEA revealed that THD-induced upregulation of genes associated with the ATF4-activated ER stress response. **D** Protein levels of p-eIF2α, ATF4, CHOP, and TRIB3 of the ER stress pathway in HT29 and LoVo cells following treatment with varying concentrations of THD for 24 h were analyzed by western blotting (left) and the corresponding quantification of these proteins is shown in the right panel. 4-PBA, an ER stress inhibitor, was treated at the concentration of 5 mM alone or in combination with 15 μM THD. The level of extracellular ICD markers including (**E**) ATP, (**F**) HMGB1, and (**G**) calreticulin (CRT) were examined using an ATP detection kit, ELISA, and flow cytometer, respectively, after 24 h of drug treatment in CRC cells SW480. Data are mean ± SD (*N* = 3). *: *p* < 0.05; **: *p* < 0.01; ***: *p* < 0.001

To identify the genes whose expressions were significantly affected by THD, next-generation sequencing (NGS) was performed using the total mRNA prepared from HT29 cells following treatment with 15 μM THD for 6 h. Interestingly, genes encoding the autophagic marker, *SQSTM1*, and ER stress marker, *TRIB3*, were among the most upregulated ones (Fig. 2A). In addition, genes encoding other components of autophagy and ER stress pathways were also found to be increased in their expression in HT29 cells due to THD treatment (Fig. 2B). Furthermore, Gene Set Enrichment Analysis (GSEA) revealed that THD-induced a gene set similar to the Activating Transcription Factor 4 (ATF4)-associated response to ER stress (Fig. 2C). Therefore, we examined the protein levels of several critical components of the ATF4-related ER stress in HT29 and LoVo CRC cells under varying THD concentrations. As shown in Fig. 2D, THD led to an increase in the protein levels of ER stress markers, including p-eIF2α, ATF4, CHOP, and TRIB3, in a dose-dependent manner in both CRC lines. A similar pattern for these markers was also observed in a time-dependent manner (Supplementary Figure S3). Activation of other ER stress pathways through ATF6 showed non-significant alteration and IRE1α showed only a slight increase after THD treatment in CRC cells (Supplementary Figure S4). In addition, not only autophagy but also ER stress was reported as one of the precedent pathways leading to ICD, three ICD markers including ATP, HMGB1, and CRT were examined with and without the presence of ER stress inhibitor 4-PBA (Figs. 2E-G). The results confirmed that THD is an ICD-inducer in CRC human cells while inhibiting ER stress could partially block this effect, indicating that THD causes the increase of ICD markers via ER stress pathway.

### Thioridazine enhances oxaliplatin-induced immunogenic cell death in CT26 mouse CRC cells

**Fig. 3:**
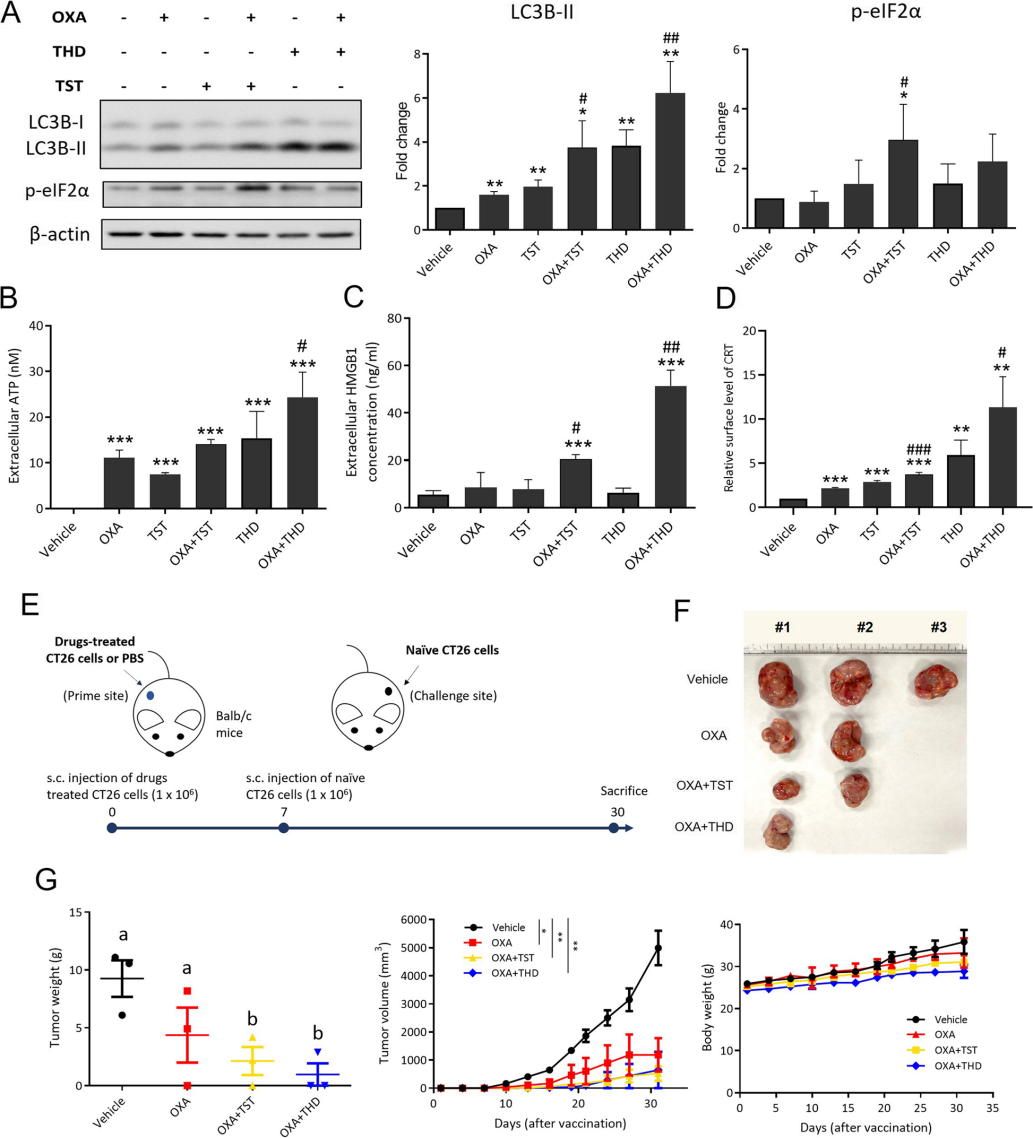
Thioridazine enhances oxaliplatin-induced immunogenic cell death in CT26 mouse CRC cells. **A** Protein levels of autophagy and ER stress markers were examined in murine CRC cells CT26 following treatment with 50 µM OXA or co-treatment of 50 µM OXA and 5 µM THD for 24 h. 5 µM thiostrepton (TST) was used as a positive control for autophagy and ICD induction. The level of extracellular ICD markers including (**B**) ATP, (**C**) HMGB1, and (**D**) calreticulin (CRT), were examined using ATP detection kit, ELISA, and flow cytometer, respectively, after 24 h of single or combined drug treatment. *: statistical significance compared to vehicle group; #: statistical significance compared to OXA treated group. *: *p* < 0.05; **: *p* < 0.01; ***: *p* < 0.001. **E** Schematic of ICD vaccination assay in Balb/c mice for 33 days. Balb/c mice were subcutaneously injected into the right flank with 1 × 10^6^ CT26 cells treated with OXA (50 μM) alone or in combination with THD (5 μM) for 24 h. One week later, each mouse was transplanted with 1 × 10^6^ naïve CT26 cells into the left flank as a challenge to examine the efficacy of vaccination. **F** Images of mice that were sacrificed, and tumors harvested from the challenge site after 30 days are shown. The mice were implanted with pre-treated CT26 cells in the prime site, which received pre-treatment with OXA alone or in combination with either TST or THD. In the vehicle group, three mice developed tumors on the challenge site. In the groups treated with OXA alone or OXA in combination with TST, two mice in each group grew tumors. However, when OXA and THD were administered together, only one mouse developed a tumor. **G** Tumor volume was calculated using the equation: 0.5 × tumor length × (tumor width).^2^ after sacrifice. Tumor growth and body weight were measured every 3 days with an electronic caliper and an electronic weight scale, respectively, until sacrifice. Data are mean ± SEM (*N* = 3). Statistical analysis was performed using one-way ANOVA with Fisher’s LSD post hoc test. *: *p* < 0.05; **: *p* < 0.01; ***: *p* < 0.001

After finding that THD to up-regulate autophagy and ER stress in human CRC cells, two pathways that have been found to be affected in various ICD inducers [21, 22], we tested whether THD could potentiate the ICD induced by OXA in CT26 mouse CRC cells by comparing its effect with that of thiostrepton (TST), a recently identified autophagy inducer [14]. OXA is prescribed for chemotherapy in CRC and it can also stimulate ICD [6, 23]. Toward this end, we observed a significant increase in LC3B-II levels in CT26 cells following treatments with 1) OXA plus TST and 2) OXA plus THD, though the effect of the latter combination was much stronger. However, a marked increase in p-eIF2α levels in these cells was only observed after they were treated with the combination of OXA plus TST (Fig. 3A). p-eIF2α is a known marker induced in both ER stress and ICD [24, 25]. We further analyzed the levels of three crucial ICD markers ATP, HMGB1, and the cell surface translocation of calreticulin (CRT) in CT26 cells following various treatments. As evidenced, THD not only enhanced OXA-induced extracellular ATP release (Fig. 3B) but also robustly increased extracellular HMGB1 (Fig. 3C) and cell surface CRT (Fig. 3D) in CT26 cells. To further assess whether the in vitro ICD of these cells induced by the combination of OXA and THD could exert an in vivo antitumor immune response, a prophylactic vaccination assay was conducted. In this experiment, we first injected drug-treated CT26 cells subcutaneously into the right flank of the immunocompetent Balb/c mice. Next, naïve CT26 cells were injected into the left flank as a challenge one week later and tumor growth from the challenge sites was monitored continuously (Fig. 3E). As can be seen in Figs. 3F & G, even though significant tumor growth suppression on the challenge sites was observed in mice receiving CT26 cells pretreated with either OXA alone or combinations of OXA + TST and OXA + THD, OXA + THD emerged as the most effective in terms of reducing tumor number, volume, and weight. The immunohistochemistry staining for T cells on challenge sites (Supplementary Figures S5A & B) demonstrated that the total T cells population and cytotoxic T cells labeled by CD3 and CD8, respectively, showed an increment in all drug treatment groups, compared to the vehicle-treated group. Interestingly, cytotoxic T cells were observed to be most abundant on the challenge site of mice receiving cancer cells treated with OXA + THD, compared to OXA or OXA + TST groups. ICD marker HMGB1 was also upregulated in all three tested treatment groups (Supplementary Figure S5A), while the changes in CRT were merely witnessed. These results suggest that THD has more potential to improve the clinical outcomes of OXA treatment in CRC cells than TST, by robustly enhancing ICD.

### The ICD effect induced by thioridazine is dependent on secretory autophagosomes

**Fig. 4:**
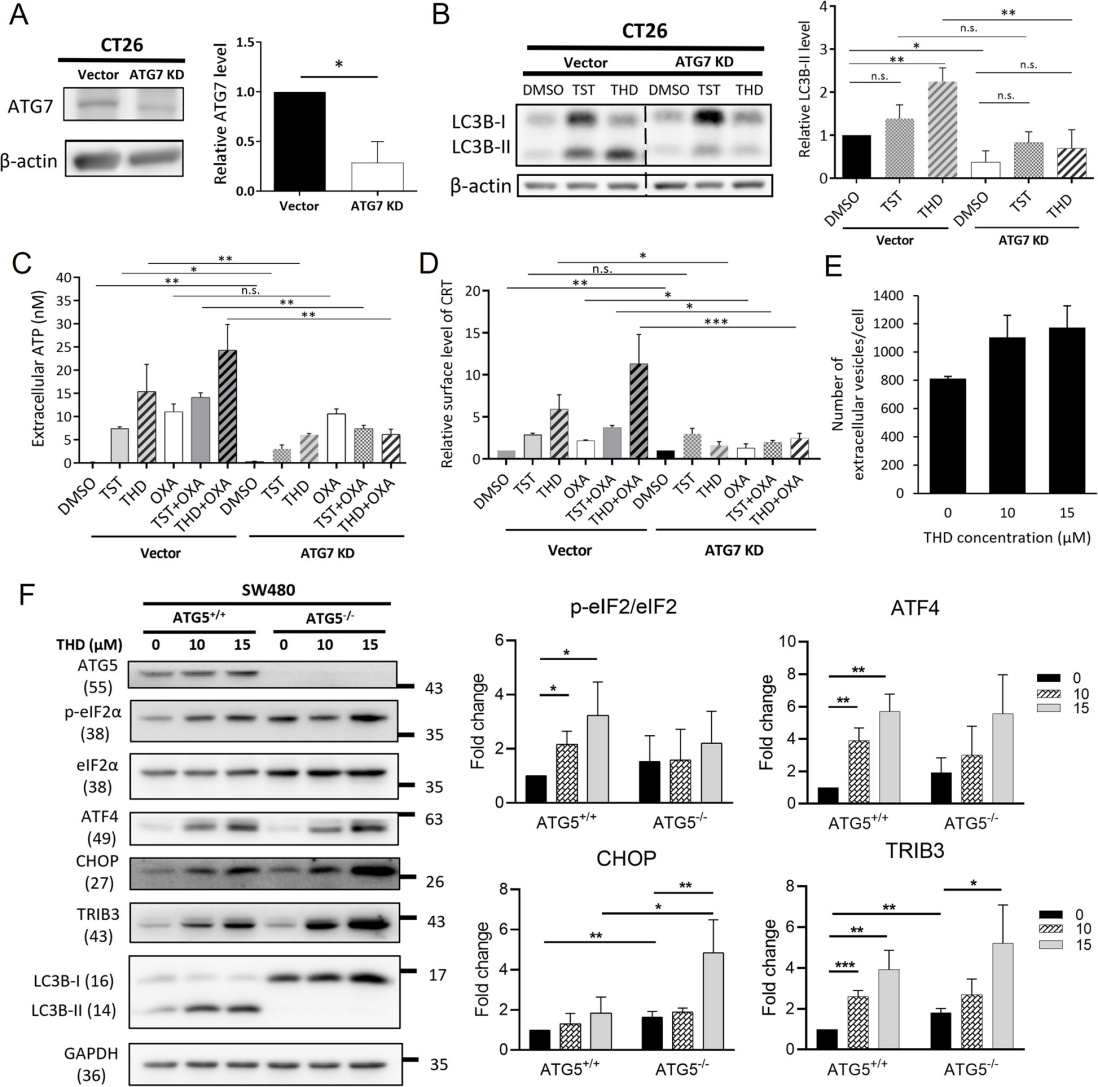
Blocking autophagosomes formation could inhibit the thioridazine-induced effect on the release of ICD markers. **A** Western blot representing knock down of ATG7 protein in CT26 cells. **B** CT26 cells with deficient ATG7 exhibited compromised autophagosomes formation under thiostrepton (TST) or THD treatment. ICD markers were detected (**C**) ATP and (**D**) calreticulin (CRT) in ATG7 KD cell following drug treatment via ATP detection assay and flow cytometry, respectively. **E** Number of secreted extracellular vesicles in HT29 cells were detected via Nanoparticles Tracking Analysis software (NanoSight NS300, Malvern Panalytical Ltd., Malvern, UK) after THD treatment for 24 h. **F** SW480 cells with intact ATG5 and ATG5 knockout were treated with THD at the concentrations of 10 and 15 μM for 24 h. The protein levels of ER stress eIF2α/ATF4/CHOP pathway components were examined using western blotting. Data are mean ± SD (*N* = 3). *: *p* < 0.05; **: *p* < 0.01; ***: *p* < 0.001

To investigate the correlation between autophagy and the ICD-inducing effect of THD in CRC, we performed a knockdown of an autophagy-initiating molecule ATG7 in CT26 cell (ATG7 KD) and examined the release of ICD markers. ATG7 depletion led to a significant reduction in the autophagy marker LC3B-II (Figs. 4A & B), potentially disrupting the ability of the cell to form autophagosomes due to TST or THD treatment. Two ICD markers, ATP (Fig. 4C) and CRT (Fig. 4D) were significantly reduced in the ATG7 KD cells, compared to the control cells following treatment with both OXA alone or in combination with either TST or THD. Besides, the nanoparticle tracking analysis in Fig. 4E accordingly demonstrated that the treatment of THD increased the number of extracellular vesicles released per cell in a dose-dependent manner. Thus, we can infer from these results that autophagosomes facilitate the extracellular excretion of ATP and the translocation of CRT to the outer side of the plasma membrane. In addition, to examine whether autophagy has a regulating role in ER stress, we used SW480 ATG5^−/−^ cells since ATG5 is an upstream molecule that triggers the formation of autophagosomes. Western blotting revealed that the ER stress-inducing effect of THD was slightly enhanced in SW480 ATG5^−/−^ cells, suggesting that ATG5 might have a mild inhibiting effect on CHOP/TRIB3 (Fig. 4F).

### The cytotoxicity of thioridazine in CRC cells

**Fig. 5:**
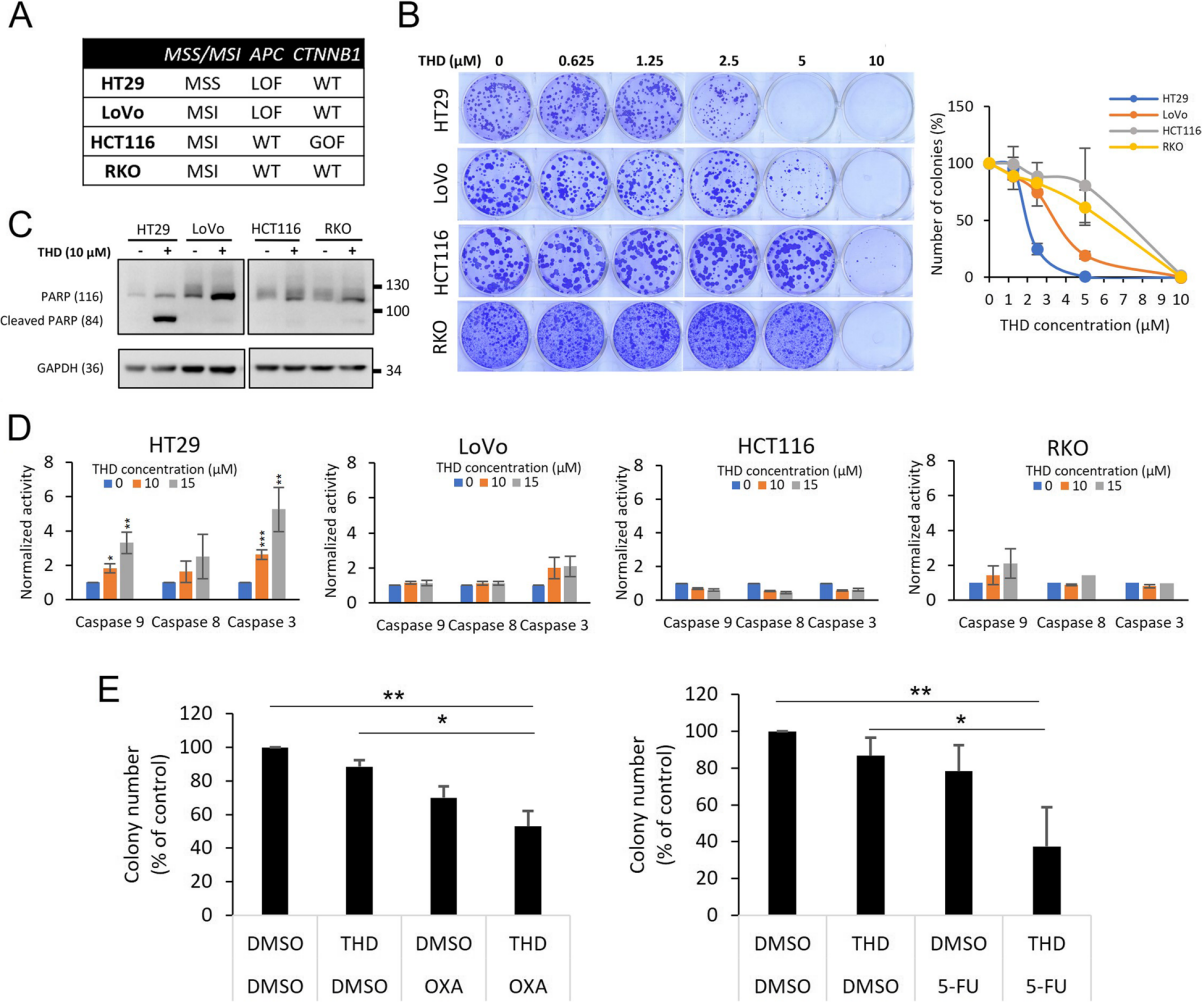
Thioridazine exhibits toxicity to CRC cells. **A** MSS/MSI classification and genetic backgrounds of HT29, LoVo, HCT116, and RKO cells representing the most frequently mutated genes in CRC based on the data obtained from DepMap database. **B** Clonogenic assay comparing the effect of THD at the dose range from 0.625 to 10 µM on different CRC cells, including HT29, LoVo, RKO, and HCT116. Cells were seeded at the density of 400 cells/well and incubated for 13–19 days. **C** The apoptosis marker, cleaved PARP was detected using western blotting in the four CRC cells following 72 h of THD treatment (10 µM). **D** Caspase 3, 9, and 8 activity assay kits were used to check the corresponding apoptosis markers, following 48 h of THD treatment at the concentration of 10 and 15 µM in HT29, LoVo, HCT116, and RKO cells. **E** The effect of sequential treatment of THD (1 µM) in combination with chemo drugs OXA (0.5 µM) or 5-FU (0.5 µM) was inspected in HT29 via clonogenic assay after 10 days of drug treatment. THD was treated from day 1 to day 3 after cell seeding, before the culture medium was replaced with medium containing chemo drug from day 4 to day 10. Data are mean ± SD (*N* = 3). *: *p* < 0.05; **: *p* < 0.01; ***: *p* < 0.001

Following confirmation of the ICD-inducing effect of THD, we questioned whether THD exhibits cytotoxicity in CRC cells, which could potentially help boost the efficacy of anticancer treatment. To investigate this, four colorectal cancer cells that carry different mutation statuses were examined: 1) HT29 and LoVo which exhibit *APC* mutation and 2) RKO and HCT116 for *APC* wild-type cells (Fig. 5A). The clonogenic assay revealed that HT29 exhibited the most sensitivity to THD, LoVo medium sensitivity, and HCT116 and RKO least sensitivity to THD (Fig. 5B). Further, western blot analysis showed that cleaved PARP, an apoptosis marker, was strongly induced in HT29 (Fig. 5C), compared to the other cell lines. Additionally, the caspase activity assay (Fig. 5D) to study the induction of apoptosis revealed that THD treatment significantly induces apoptosis in HT29, which was higher than LoVo, HCT116, and RKO. Furthermore, since the combination of the targeted drug along with the currently used chemotherapy is essential for clinical trials, our preliminary data showed that the sequential combination of THD and OXA or 5-FU in HT29 cells has a synergistic effect in preventing colony formation (Fig. 5E). Therefore, it might be concluded that THD exhibits an anticancer effect in CRC, especially toward cells carrying *APC* mutation. Further investigation in the Wnt/β-catenin pathway indicated that THD suppressed the downstream of this signaling pathway including the promoter TCF/LEF (Supplementary Figure S6A) and the protein level of c-Myc, cyclin D1, and survivin (Supplementary Figures S6B & C) in HT29 cells.

## Discussion

In this study, we have found that THD, a repurposed drug has the potential to be used for CRC treatment since 1) THD induces ER stress through eIF2α/ATF4/CHOP axis and secretory autophagy 2) enhances the ICD-inducing effect of chemotherapy and 3) induce apoptosis selectivity toward cells that exhibit Wnt/β-catenin activation and belong to the MSS classification.

It has been reported that autophagy inducers could potentially stimulate ICD through the release of ATP in autophagosomes [16]. Thus, to find a potential autophagy inducer that could also cause ICD, we performed bioinformatics analysis using the CLUE database to obtain a candidate drug list. The Connectivity Map is a database of compounds against transcriptional expression profiles that can help users to find drug-disease relationships and the mechanism of action of drugs [26]. To obtain possible autophagy inducers, gene profiles from HT29 cells treated with TST, sirolimus, torin-1, and simvastatin were used as references. Various antipsychotic agents with previously reported potential benefits for cancer treatment were observed in the common compound list [27]. Out of these, the dopamine receptor antagonist THD was selected as it showed the highest toxicity toward CRC cells. THD is a phenothiazine derivative traditionally prescribed for schizophrenia and psychosis. There are several studies reporting the anticancer effects of THD in various cancer types such as glioblastoma, breast, ovarian, and endometrial cancer [17, 21, 28–31]. However, the molecular mechanism of THD in cancer remains ambiguous. In addition, THD has two enantiomers: the S-enantiomer which binds strongly to dopamine D1 receptors, and the R-enantiomer which binds to D2 receptors [32]. In fact, patients were administered with the racemic form of THD but were subsequently withdrawn owing to its tremendous side effects such as allergic reactions, trouble controlling body movements, and neuroleptic malignant syndrome. These side effects are believed to be caused mainly by the S-enantiomer since it was observed to cause higher toxicity and catalepsy in rats at large doses than the R-enantiomer [32]. Therefore, in the future, clinical administration of a single enantiomer of THD, rather than the racemic mixture, would provide a safer treatment remedy for cancer patients.

The autophagy-inducing effect of THD was observed to be time-dependent due to the long-term accumulation of autophagosomal markers LC3B-II and p62. Additionally, while the treatment of the early-stage autophagy inhibitor 3-MA was able to partially reduce the effect of THD-induced autophagy, the late-stage autophagy inhibitor BafA1 did not have any significant effect on THD. This suggests that THD might not only increase the transcription of autophagy markers but could also play a role in preventing the degradation of autophagosomes, possibly inducing subsequent secretory autophagosomes.

NGS analysis revealed that THD could induce ER stress response along with autophagy in CRC cells, which we have validated using western blotting and show that THD significantly up-regulates eIF2α/ATF4/CHOP pathway, rather than ATF6 or IRE1α pathways (Fig. 2 & Supplementary Figure S4). THD has previously been reported to induce ER stress markers in glioblastoma, cervical cancer, and leukemia cells [17, 30, 33]. In this study, we have further elucidated the correlation between THD-induced ER stress, autophagy, and the ICD effect. In addition, according to a previous report ER stress induces autophagy [31] to digest cellular waste as a compensation mechanism for the unfolded protein response. However, HT29 cells treated with THD following RNAi depletion for CHOP autophagy markers were unaffected (Supplementary Figure S7). In addition, the accumulation of LC3B-II distinctly occurred at an early stage (3 h post-THD treatment), whereas the accumulation of ER stress markers such as ATF4, p-eIF2α, CHOP, and TRIB3 appeared 24 h post-THD treatment at the same dose, indicating that autophagy might not be downstream of ER stress in THD treated conditions. Instead, as shown in Fig. 4E, eliminating autophagy upstream ATG5 might have the ability to slightly facilitate the downstream of ER stress including CHOP and TRIB3. According to a previously published report, inhibiting autophagy in HT29 could sensitize these cells to chemoradiation [34], indicating that autophagy might play a protective role in CRC. THD, however, induces excessive autophagy in HT29 up to 48 h. Combined with the parallel effect on ER stress induction, THD could potentially contribute to tumor suppression via ICD.

In our study, we show that the single treatment of OXA boosts the release of ICD markers ATP, HMGB1, and CRT since OXA has been known as an ICD inducer. Additionally, the combination of OXA with THD significantly improves the OXA-induced ICD effect, as observed in both in vitro and in vivo experiments. The contribution of THD to the ICD effect could be attributed not only to the increment of ER stress markers but also to the secretory autophagosomes since the extracellular secretion of ATP and HMGB1 were reported to be autophagy-dependent [35]. To support this, the results shown in Figs. 4C & D support the idea that ICD marker expression is dependent on the formation of autophagosomes since the knockdown of autophagy component ATG7 showed a significant decrease in the release of ATP and CRT from CT26 cells. CT26 xenograft model could demonstrate the vaccine-like effect of OXA + THD in the right flank of the mice, preventing the tumor formation of naïve CT26 injected in the left flank 7 days later. This experimental model for the ICD effect was reported in previous studies [23, 36]. ICD inducers such as doxorubicin and mitoxantrone-treated cells have been reported to induce the death of murine cells with an outcome where 80% of mice became tumor-free [9]. The ICD-enhancing effect supplementary to chemotherapy was demonstrated in another study wherein trifluridine/tipiracil, a drug for metastatic colon cancer, was able to induce in vitro ICD and facilitate the activation of CD8^+^ T cells when combined with OXA in xenograft model of SW620, Caco-2, and Colo-320 [37].

Among the four tested human CRC cells, HT29 exhibited the most sensitivity to treatment THD treatment as evidenced by the activation of apoptotic cell death and reduced colony-forming ability. Interestingly, HT29 belongs to the MSS group of CRC, the group that is non-responsive to immunotherapy [7], whereas LoVo, HCT116, and RKO are members of the MSI group (Fig. 5A). Furthermore, the mouse CRC cell line CT26 shares the same group as HT29 [38]. Therefore, we propose a possible application of THD for the stratification of MSS CRC patients in clinical settings.

THD was observed to suppress the activation of downstream targets of the Wnt/β-catenin pathway (Supplementary Figure S6). Although the protein expression level of β-catenin and the active form phosphorylated β-catenin Ser675 showed no significant differences, the TCF/LEF reporter activity was significantly suppressed. Furthermore, c-Myc, cyclin D1, and surviving, the downstream targets of the Wnt/ β-catenin pathway exhibited a decrease in a dose-dependent manner, 24 h post-THD treatment in HT29 cells. This data showed a contrasting mechanism of THD in down-regulating the Wnt/β-catenin pathway, compared to glioblastoma, where the active β-catenin was degraded by THD [18]. In addition, a previous study reported that nuclear β-catenin could act as a suppressor of p62 transcription [39], thus suggesting that the down-regulation of the Wnt pathway could contribute to the upregulation of p62 and autophagosome formation. Tumors with active Wnt/β-catenin signaling have been reported to have low infiltration of immune cells. Additionally, a preclinical study suggested that the inhibition of this pathway could sensitize CRC tumors to immunotherapy [40]. Therefore, THD is a promising candidate to be combined with not only chemotherapy but also immunotherapy to further improve the outcome of combination treatment in CRC.

## Conclusion

**Fig. 6:**
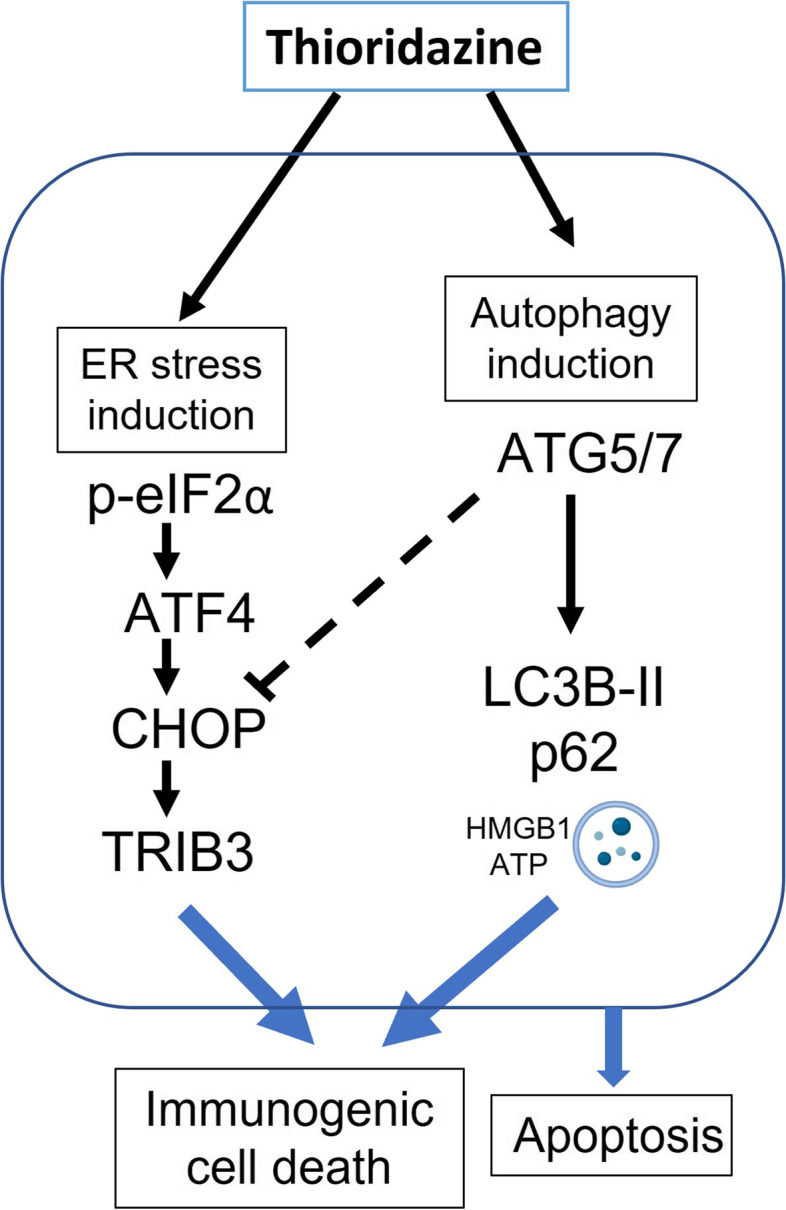
Proposed mechanism of thioridazine in CRC. A model to propose the anticancer effect of THD in CRC through the enhancement of the autophagy and ER stress responses, ultimately induce apoptosis and enhance chemo-induced immunogenic cell death

In conclusion, we report a novel repurposed role for THD in CRC, in addition to anticancer stem cell inhibition and antipsychotic role. This finding delineates the moonlighting role of THD in inducing secretory autophagy and ER stress ultimately leading to apoptosis and enhanced chemotherapy-induced ICD (Fig. 6). Therefore, combination chemotherapy such as oxaliplatin with THD might enhance the antitumor in CRC patients.

## Supplementary Information

**Additional file 1: Supplementary Figure S1.** Comparison of the cytotoxicity of dopamine receptor antagonists in CRC cells. Seven CRC cells were treated with thioridazine, prochlorperazine, and trifluoperazine for 72 h. The cytotoxicity of the drugs was measured via SRB assay, revealing that thioridazine is the most potent against CRC cells. **Supplementary Figure S2.** Thioridazine induces autophagy in various human CRC cells.CRC cells including LoVo and RKO were treated with THD at the concentrations of 10 and 15 μM for 24 h before being lysed and detected for autophagy marker LC3B-II by western blotting.Protein levels of LC3B-II in LoVo cells following 15 µM THD treatment in the absence or presence of 3-MAand BafA1for 6 h were analyzed by western blotting. GAPDH was used as the internal control inand. **Supplementary Figure S3.** Thioridazine induces ER stress in a time-dependent manner. HT29 cells were treated with THD at the concentration of 15 µM for a period of time from 3 to 48 h. Protein expression levels of ER stress pathway components were examined using western blotting. *: *p* < 0.05; **: *p* < 0.01; ***: *p* < 0.001. **Supplementary Figure S4.** THD exhibits no significant effect on ATF6 and IRE1α pathways.The protein expression level of ATF6 after THD treatment at the concentration of 15 μM at different time points.The protein expression level of IRE1α was examined after THD treatment at the doses of 10 and 15 μM for 24 h. **Supplementary Figure S5:** ICD markers on the challenge site tumors via immunohistochemistry staining.Tumors on the challenge site of the experiment from Figure 3E were collected, embedded in paraffin, and sliced for IHC staining. T cell markers CD3 and CD8, and ICD markers HMGB1 and CRT were examined and quantified by H-score. Data are mean ± SD. Since the number of tumors was small, statistical analysis was not applicable. The H-score of each tumor slice was calculated by the formula: H-score = Σ. Representative images of intensity score.Representative images of cytotoxic T cell staining via CD8 marker. Brown: CD8, blue: nucleus. Red square: positions of the zoom-in images. Scale bar of upper panel: 9 mm, and lower panel: 200 μm. **Supplementary Figure S6.** Thioridazine decreases Wnt/β-catenin pathway activity.TCF/LEF reporter assay was measured in HT29, LoVo, and HCT116 after 24 and 48 h of THD treatment at the concentration of 10 and 15 µM.Western blot and quantification displaying protein expressions of Wnt/β-catenin pathway components in HT29 following THD treatment for 24 h at various doses from 2.5 to 15 µM. Data are mean ± SD.Representative images and quantifications of immunofluorescence staining showing the effect of THD on nuclear c-Myc and β-catenin after 24 h of THD treatment at 15 µM. Data are mean ± SD. Scale bar: 10 µm. *: *p* < 0.05; **: *p* < 0.01; ***: *p* < 0.001. **Supplementary Figure S7.** The knockdown of CHOP-encoding gene DDIT3 has no significant effect on thioridazine-induced autophagy. Lentivirus mediated shRNA knockdown of the DDIT3 gene was performed in HT29 cells was knocked down via. After 24 h of incubation with lentivirus, cells were selected with puromycin for 48 h. Untransfected, empty vector transfected, and shRNA transfected cells were treated with THD at the concentration of 15 μM for 24 h.

## Data Availability

All data generated or analyzed during this study are included in this published article.

## Abbreviations

CRC: Colorectal cancer
ICD: Immunogenic cell death
THD: Thioridazine
ER: Endoplasmic reticulum
OXA: Oxaliplatin
APC: Adenomatous Polyposis Coli
ICIs: Immune checkpoint inhibitors
MSS: Microsatellite stability
MSI: Microsatellite instability
DAMPs: Damage-associated molecular patterns
CRT: Calreticulin
ATP: Adenosine triphosphate
HMGB1: High mobility group box 1
TST: Thiostrepton
TCF/LEF: T-cell factor/lymphoid enhancer factor
3-MA: 3-Methoxyamphetamine
BafA1: Bafilomycin A1
NGS: Next-generation sequencing
5-FU: 5-Fluorouracil
