# Supplementary material for: Repurposing thioridazine for inducing immunogenic cell death in colorectal cancer via eIF2α/ATF4/CHOP and secretory autophagy pathways
Source: Cell Commun Signal. 2023 Jul 24;21:184. doi: 10.1186/s12964-023-01190-5 (PMC10364410; doi:10.1186/s12964-023-01190-5)
Supplement: Supplementary file 2 — Additional file 1: Supplementary Figure S1. Comparison of the cytotoxicity of dopamine receptor antagonists in CRC cells. Seven CRC cells were treated with thioridazine, prochlorperazine, and trifluoperazine for 72 h. The cytotoxicity of the drugs was measured via SRB assay, revealing that thioridazine is the most potent against CRC cells. Supplementary Figure S2. Thioridazine induces autophagy in various human CRC cells.CRC cells including LoVo and RKO were treated with THD at the concentrations of 10 and 15 μM for 24 h before being lysed and detected for autophagy marker LC3B-II by western blotting.Protein levels of LC3B-II in LoVo cells following 15 µM THD treatment in the absence or presence of 3-MAand BafA1for 6 h were analyzed by western blotting. GAPDH was used as the internal control inand. Supplementary Figure S3. Thioridazine induces ER stress in a time-dependent manner. HT29 cells were treated with THD at the concentration of 15 µM for a period of time from 3 to 48 h. Protein expression levels of ER stress pathway components were examined using western blotting. *: p < 0.05; **: p < 0.01; ***: p < 0.001. Supplementary Figure S4. THD exhibits no significant effect on ATF6 and IRE1α pathways.The protein expression level of ATF6 after THD treatment at the concentration of 15 μM at different time points.The protein expression level of IRE1α was examined after THD treatment at the doses of 10 and 15 μM for 24 h. Supplementary Figure S5: ICD markers on the challenge site tumors via immunohistochemistry staining.Tumors on the challenge site of the experiment from Figure 3E were collected, embedded in paraffin, and sliced for IHC staining. T cell markers CD3 and CD8, and ICD markers HMGB1 and CRT were examined and quantified by H-score. Data are mean ± SD. Since the number of tumors was small, statistical analysis was not applicable. The H-score of each tumor slice was calculated by the formula: H-score = Σ. Representative images of intensity score.Representative [file 12964_2023_1190_MOESM1_ESM.docx]

**Supplementary data**

**Repurposing thioridazine for inducing immunogenic cell death in colorectal cancer via eIF2α/ATF4/CHOP and secretory autophagy pathways**

Thu-Ha Tran, Ming Kao, Hsiao-Sheng Liu, Yi-Ren Hong, Yeu Su^*^, Chi-Ying F. Huang^*^


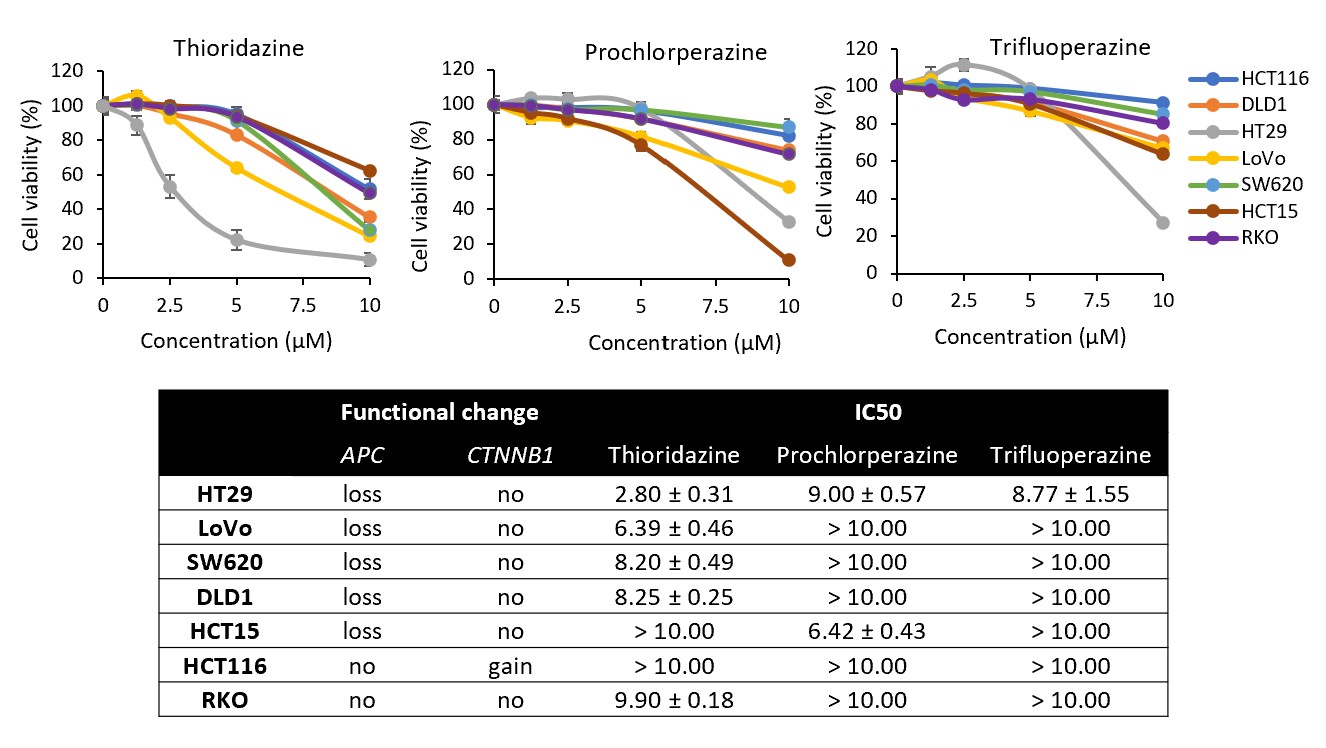


**Supplementary Figure S1: Comparison of the cytotoxicity of dopamine receptor antagonists in CRC cells.** Seven CRC cells were treated with thioridazine, prochlorperazine, and trifluoperazine for 72 h. The cytotoxicity of the drugs was measured via SRB assay, revealing that thioridazine is the most potent against CRC cells.


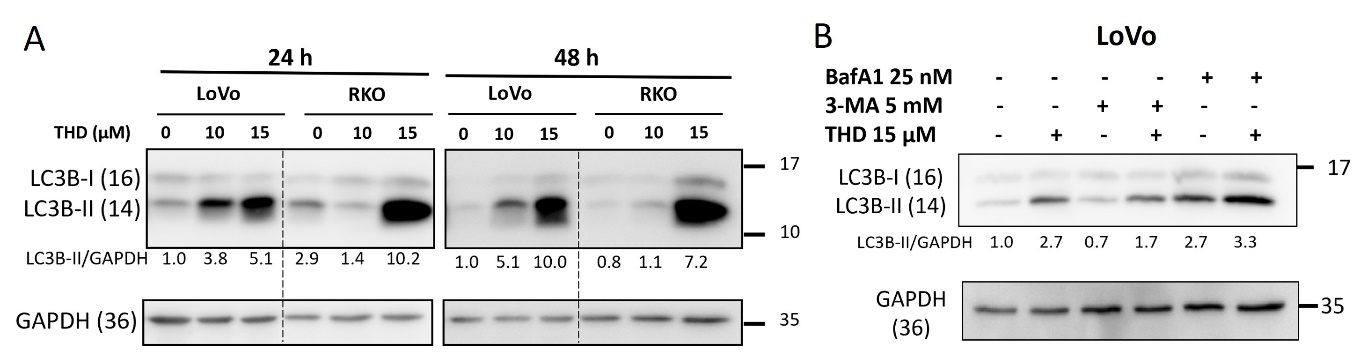


**Supplementary Figure S2: Thioridazine induces autophagy in various human CRC cells**. (A) CRC cells including LoVo and RKO were treated with THD at the concentrations of 10 and 15 μM for 24 h before being lysed and detected for autophagy marker LC3B-II by western blotting. (B) Protein levels of LC3B-II in LoVo cells following 15 µM THD treatment in the absence or presence of 3-MA (5 mM) and BafA1 (25 nM) for 6 h were analyzed by western blotting. GAPDH was used as the internal control in (A) and (B).


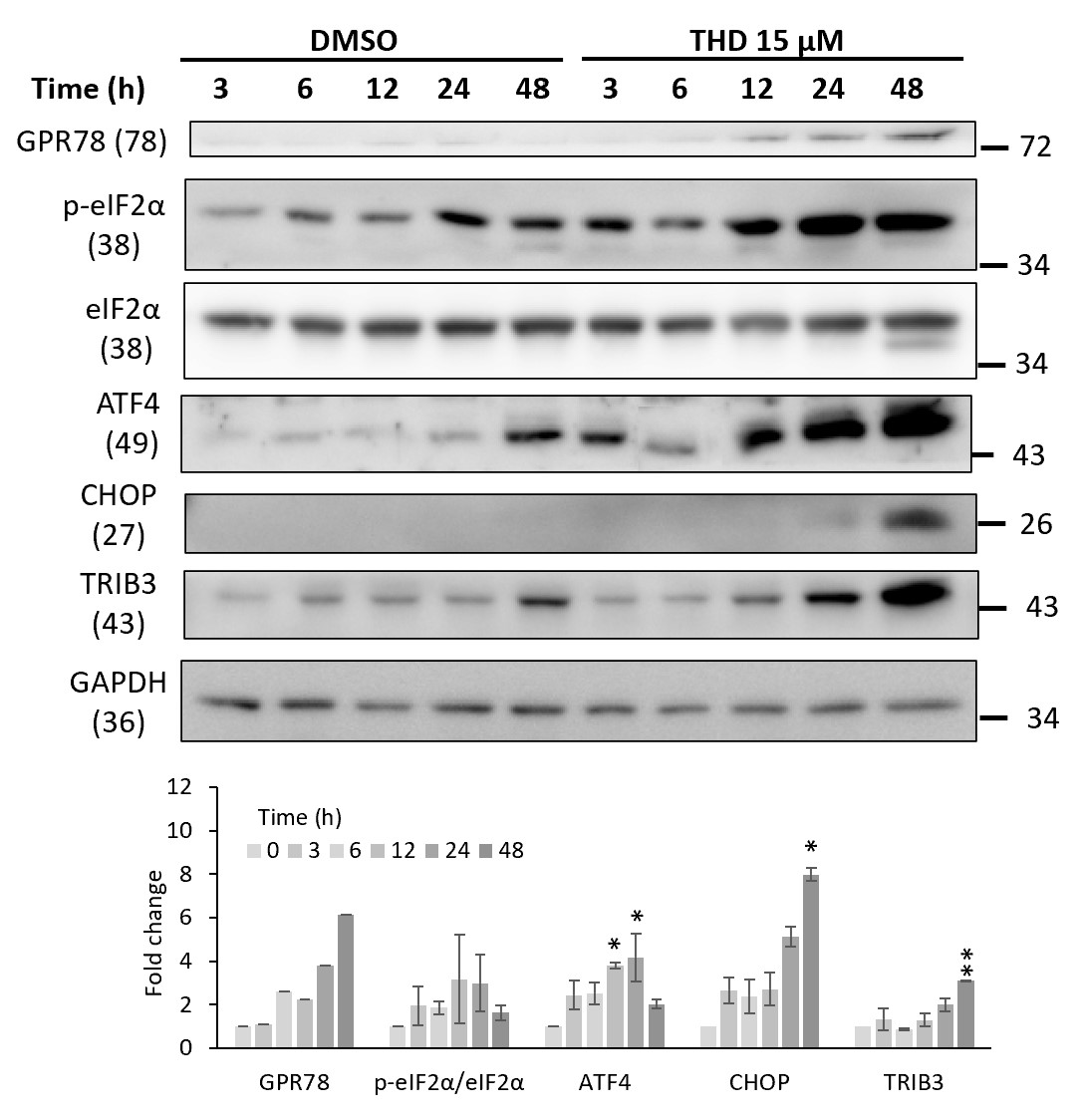
**Supplementary Figure S3: Thioridazine induces ER stress in a time-dependent manner.** HT29 cells were treated with THD at the concentration of 15 µM for a period of time from 3 to 48 h. Protein expression levels of ER stress pathway components were examined using western blotting. *: *p* < 0.05; **: *p* < 0.01; ***: *p* < 0.001.


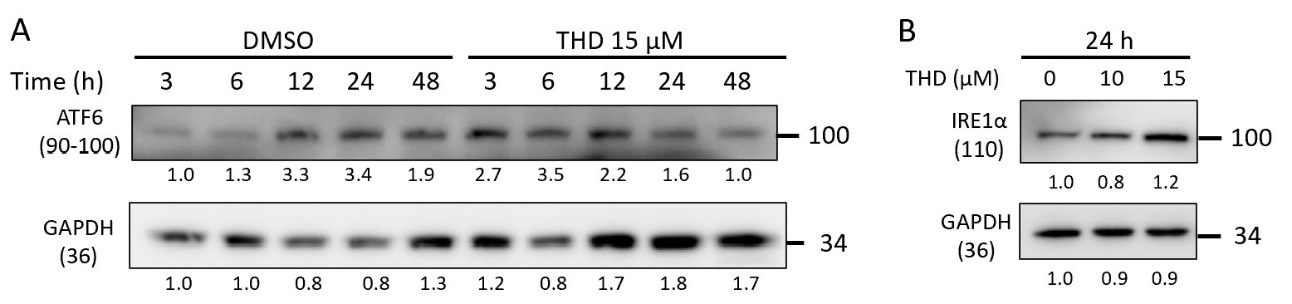


**Supplementary Figure S4: THD exhibits no significant effect on ATF6 and IRE1α pathways.** (A) The protein expression level of ATF6 after THD treatment at the concentration of 15 μM at different time points. (B) The protein expression level of IRE1α was examined after THD treatment at the doses of 10 and 15 μM for 24 h.


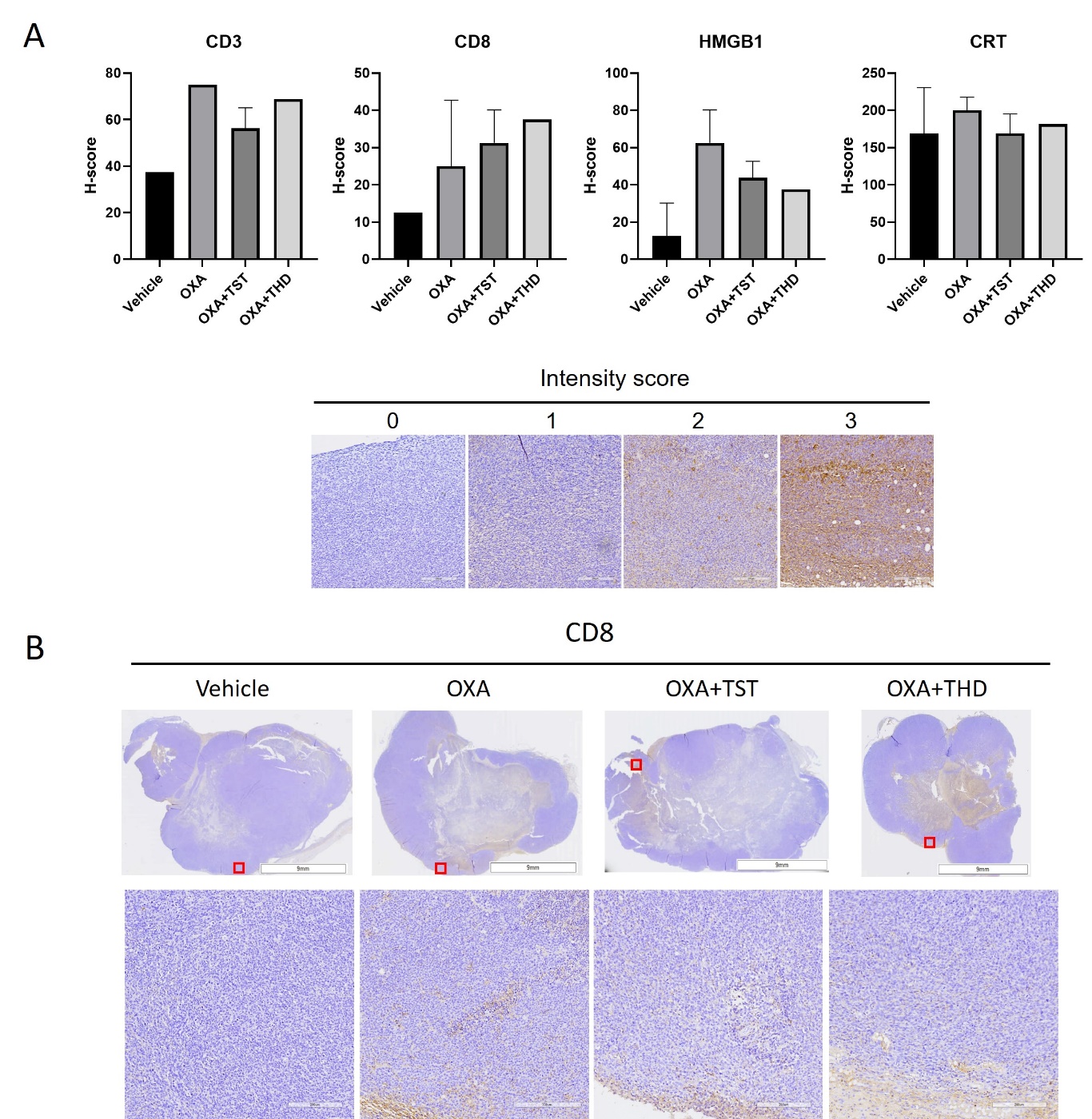


**Supplementary Figure S5: ICD markers on the challenge site tumors via immunohistochemistry staining.** (A) Tumors on the challenge site of the experiment from Figure 3E were collected, embedded in paraffin, and sliced for IHC staining. T cell markers CD3 and CD8, and ICD markers HMGB1 and CRT were examined and quantified by H-score (upper panel). Data are mean ± SD. Since the number of tumors was small, statistical analysis was not applicable. The H-score of each tumor slice was calculated by the formula: H-score = Σ (intensity × percentage). Representative images of intensity score (lower panel). (B) Representative images of cytotoxic T cell staining via CD8 marker. Brown: CD8, blue: nucleus. Red square: positions of the zoom-in images. Scale bar of upper panel: 9 mm, and lower panel: 200 μm.


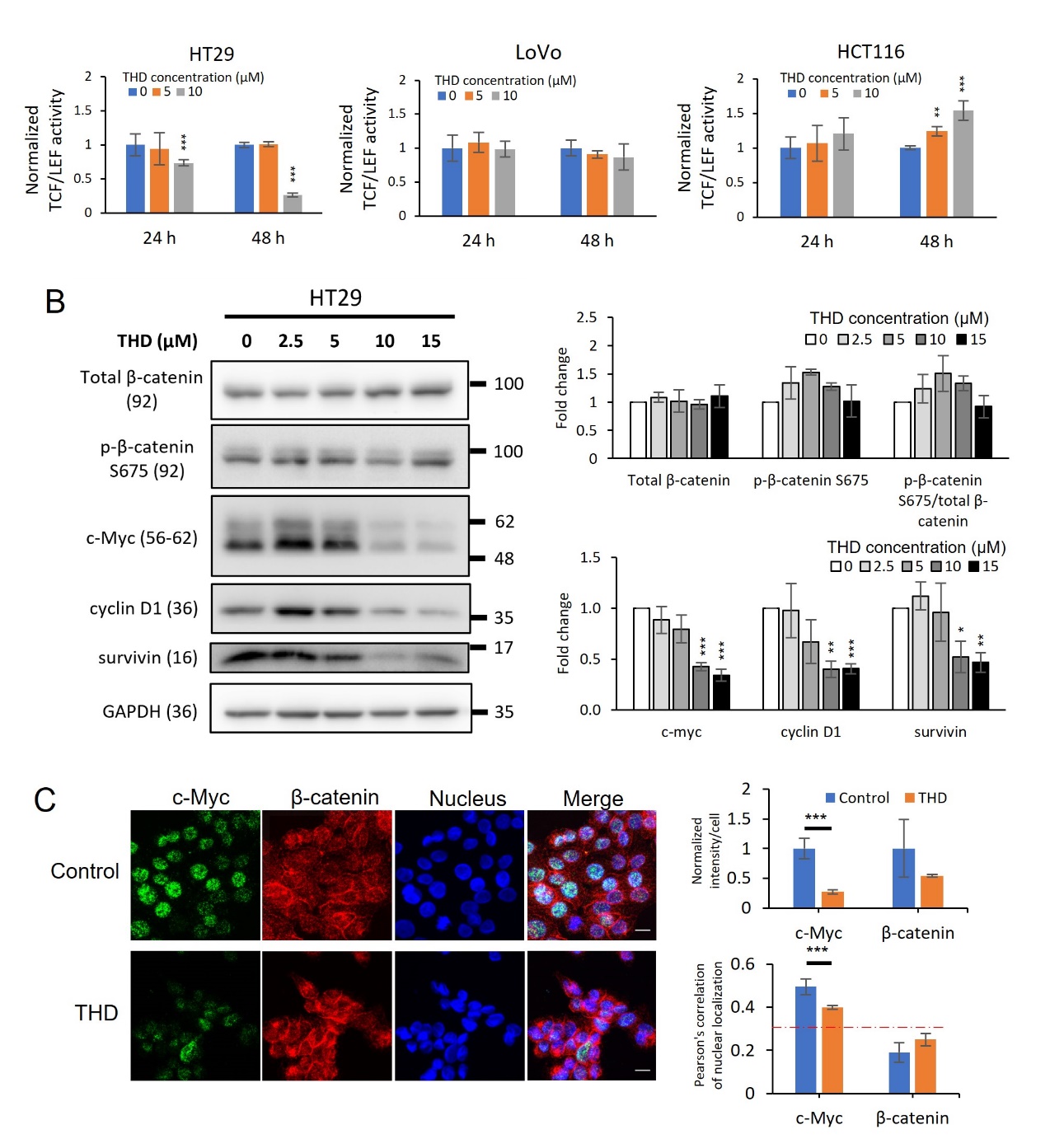
**Supplementary Figure S6: Thioridazine decreases Wnt/β-catenin pathway activity.** (A) TCF/LEF reporter assay was measured in HT29, LoVo, and HCT116 after 24 and 48 h of THD treatment at the concentration of 10 and 15 µM. (B) Western blot and quantification displaying protein expressions of Wnt/β-catenin pathway components in HT29 following THD treatment for 24 h at various doses from 2.5 to 15 µM. Data are mean ± SD (N = 3). (C) Representative images and quantifications of immunofluorescence staining showing the effect of THD on nuclear c-Myc and β-catenin after 24 h of THD treatment at 15 µM. Data are mean ± SD (N = 10). Scale bar: 10 µm. *: *p* < 0.05; **: *p* < 0.01; ***: *p* < 0.001.


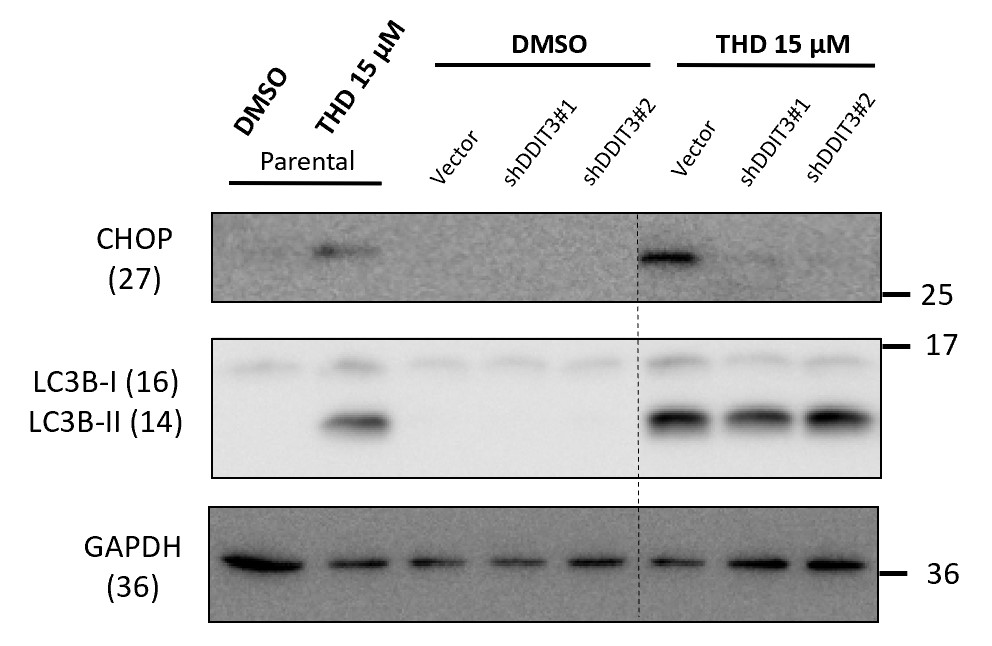
**Supplementary Figure S7: The knockdown of CHOP-encoding gene DDIT3 has no significant effect on thioridazine-induced autophagy.** Lentivirus mediated shRNA knockdown of the DDIT3 gene was performed in HT29 cells was knocked down via. After 24 h of incubation with lentivirus, cells were selected with puromycin for 48 h. Untransfected, empty vector transfected, and shRNA transfected cells were treated with THD at the concentration of 15 μM for 24 h.
